# Tumor Suppression by Anti-Fibroblast Activation Protein Near-Infrared Photoimmunotherapy Targeting Cancer-Associated Fibroblasts

**DOI:** 10.3390/cancers16020449

**Published:** 2024-01-20

**Authors:** Raisa A. Glabman, Colleen P. Olkowski, Hannah A. Minor, Laura L. Bassel, Noemi Kedei, Peter L. Choyke, Noriko Sato

**Affiliations:** 1 Molecular Imaging Branch, Center for Cancer Research, National Cancer Institute, National Institutes of Health, Bethesda, MD 20892, USA; glabmanr@msu.edu (R.A.G.); colleen.olkowski@nih.gov (C.P.O.); hminortx@gwmail.gwu.edu (H.A.M.); pchoyke@mail.nih.gov (P.L.C.); 2 Department of Pathobiology and Diagnostic Investigation, College of Veterinary Medicine, Michigan State University, East Lansing, MI 48824, USA; 3 Center for Advanced Preclinical Research, Frederick National Laboratory for Cancer Research, National Cancer Institute, National Institutes of Health, Frederick, MD 21701, USA; laura.bassel@nih.gov; 4 Collaborative Protein Technology Resources, Office of Science and Technology Resources, Center for Cancer Research, National Cancer Institute, National Institutes of Health, Bethesda, MD 20892, USA; kedein@mail.nih.gov

**Keywords:** fibroblast activation protein, cancer-associated fibroblast, near-infrared photoimmunotherapy, tumor microenvironment, cancer therapy

## Abstract

**Simple Summary:**

Cancer-associated fibroblasts (CAFs) are a key pro-tumorigenic cell type in the tumor microenvironment; however, treatments targeting these cells have shown limited clinical efficacy. In this study, we examined the therapeutic efficacy of CAF depletion using near-infrared photoimmunotherapy (NIR-PIT), a novel technique that selectively targets and depletes specific cells within a tumor. In two tumor models (lung, mammary) with immune-competent mice, selective depletion of fibroblast activation protein (FAP)-expressing cells using NIR-PIT induced interferon-gamma production in tumor-infiltrating CD8 T and natural killer cells and successfully suppressed tumor growth and reduced lung metastasis in the mammary model. These findings highlight the role of CAFs in supporting tumor growth and introduce a promising therapeutic approach for selectively eliminating immunosuppressive FAP^+^ cells within the tumor microenvironment.

**Abstract:**

Cancer-associated fibroblasts (CAFs) constitute a prominent cellular component of the tumor stroma, with various pro-tumorigenic roles. Numerous attempts to target fibroblast activation protein (FAP), a highly expressed marker in immunosuppressive CAFs, have failed to demonstrate anti-tumor efficacy in human clinical trials. Near-infrared photoimmunotherapy (NIR-PIT) is a highly selective tumor therapy that utilizes an antibody-photo-absorbing conjugate activated by near-infrared light. In this study, we examined the therapeutic efficacy of CAF depletion by NIR-PIT in two mouse tumor models. Using CAF-rich syngeneic lung and spontaneous mammary tumors, NIR-PIT against FAP or podoplanin was performed. Anti-FAP NIR-PIT effectively depleted FAP^+^ CAFs, as well as FAP^+^ myeloid cells, and suppressed tumor growth, whereas anti-podoplanin NIR-PIT was ineffective. Interferon-gamma production by CD8 T and natural killer cells was induced within hours after anti-FAP NIR-PIT. Additionally, lung metastases were reduced in the treated spontaneous mammary cancer model. Depletion of FAP^+^ stromal as well as FAP^+^ myeloid cells effectively suppressed tumor growth in bone marrow chimeras, suggesting that the depletion of both cell types in one treatment is an effective therapeutic approach. These findings highlight a promising therapy for selectively eliminating immunosuppressive FAP^+^ cells within the tumor microenvironment.

## 1. Introduction

The tumor microenvironment (TME) includes cell subtypes that contribute to both immune evasion and immune suppression [1]. Among them, cancer-associated fibroblasts (CAFs) constitute a key cellular component of the tumor stroma, serving several immunosuppressive functions within the TME [2]. Pro-tumorigenic roles of CAFs include remodeling the extracellular matrix (ECM), suppressing anti-tumor immunity, and aiding tumor cells in resistance to therapy [2], supporting neoplastic cells throughout the disease spectrum, from early seeding to metastasis. Depleting CAFs as a therapeutic strategy has the potential to reduce angiogenesis, epithelial–mesenchymal transition, and immune evasion [3]. Moreover, CAFs are more genetically stable compared with neoplastic cells and less likely to develop resistant phenotypes caused by high mutation rates and clonal selection. Additionally, they maintain epigenetic differences compared with normal resting stromal cells [4,5,6]. However, targeting CAFs presents several challenges owing to their diverse origins, plasticity, expression of heterogeneous markers, and phenotypic variation across cancer and tissue types [7].

Fibroblast activation protein (FAP), a type II transmembrane serine protease family glycoprotein [8], is commonly expressed by CAFs. FAP is minimally expressed by fibroblasts in the resting state [9] but is highly upregulated by CAFs in cancer as well as other fibroproliferative diseases (e.g., idiopathic pulmonary fibrosis, hepatic fibrosis, rheumatoid arthritis, and myocardial infarction) [10,11]. High FAP expression has been correlated to higher tumor grade, high recurrence rates, and poor survival across a wide range of human cancers, including breast [12,13,14], oral squamous cell carcinoma [15], gastric [16,17], renal [18], colorectal [19,20], lung [11,21], ovarian [22], pancreatic [23,24], and melanoma [25,26]. FAP promotes tumor growth by promoting angiogenesis and ECM remodeling [27] and facilitates the progression of tumors by suppressing the anti-cancer immune response [28,29]. FAP is upregulated in vitro and in vivo by transforming growth factor (TGF)-β and interleukin (IL)-1β [30]. Despite abundant evidence that FAP is critical in the TME and preclinical FAP^+^ cell inhibition using small molecule inhibitors, pharmacologic inhibitors, or FAP-selective protoxins [1,21,31], FAP-targeted therapies have not shown efficacy in early-phase clinical trials [32,33,34,35].

Near-infrared photoimmunotherapy (NIR-PIT) is a novel technique to selectively target and deplete specific cells locally within a tumor. Using an antibody conjugated to the phthalocyanine dye, IR700, target cells rapidly undergo necrosis after exposure to NIR light [36,37]. Several clinical trials using epidermal growth factor receptor (EGFR)-targeted NIR-PIT in head and neck cancers (http://clinicaltrials.gov/ (accessed on 28 September 2023) Identifier: NCT02422979) are underway. NIR-PIT for advanced head and neck cancers has been approved for clinical use in Japan under the tradename Alluminox™ [38] (Rakuten Medical Inc. https://rakuten-med.com/us/ (accessed on 28 September 2023)). While the current NIR-PIT platforms directly target tumor antigens, it is also possible to target and deplete immunosuppressive cells in the TME. For instance, CD4^+^CD25^+^ regulatory T (Treg) cells have been locally depleted using NIR-PIT to augment the anti-tumor immune response [39]. In a similar manner, fibroblasts can be selectively targeted using anti-FAP NIR-PIT [40,41,42]. FAP-transduced fibroblast cells mixed with cancer cells have been treated with NIR-PIT, resulting in tumor growth inhibition [42]. 

In this study, we used a syngeneic mouse lung cancer model and a spontaneous mammary tumor model, both containing physiologically differentiated CAFs in the TME, and investigated the therapeutic effects of CAF-targeted NIR-PIT. We demonstrate that anti-FAP NIR-PIT can effectively deplete endogenous CAFs in the TME, induce anti-tumor effector cell activation and IFN-γ production, and suppress tumor growth. 

## 2. Materials and Methods

### 2.1. Cell Culture

Murine cell lines NIH3T3 fibroblasts, LL/2 Lewis lung carcinoma, MOC2 squamous cell carcinoma cells, EL-4 T lymphoblast, EO771 mammary carcinoma, 4T1 mammary carcinoma, and PAN02 pancreatic adenocarcinoma cells were purchased from American Type Culture Collection (ATCC, Manassas, VA, USA). MC38 murine colon adenocarcinoma cells were purchased from Kerafast (Shirley, MA, USA). NIH3T3 cells were maintained in DMEM medium (Thermo Fisher Scientific, Waltham, MA, USA), and other cells were cultured in RPMI1640 (Thermo Fisher Scientific), supplemented with 10% fetal calf serum (FCS, GeminiBio, West Sacramento, CA, USA), 100 IU/mL penicillin/streptomycin (Thermo Fisher Scientific), and 0.05 mM 2-mercaptoethanol (Sigma-Aldrich, St. Louis, MO, USA). All cell lines tested negative for *Mycoplasma* via polymerase chain reaction (PCR) using a Mycoplasma PCR detection kit (ABM) (Frederick National Laboratory for Cancer Research, Frederick, MD, USA). All cells were cultured in a humidified incubator at 37 °C with 5% CO_2_. 

### 2.2. Conjugation of Anti-FAP and Anti-PDPN Antibodies with IR700 Dye

Conjugation of IR700 with monoclonal antibodies was performed according to previous reports [37]. Briefly, 500 mg of anti-FAP (Clone 983802; R&D Systems, Minneapolis, MN, USA) or anti-podoplanin (PDPN) (Clone 8.1.1; BioXCell, Lebanon, NH, USA) antibody was incubated with molar excess of IR700 (LI-COR Biosciences (Lincoln, NE, USA) and Rakuten Medical (San Diego, CA, USA)) in 0.1 mol/L sodium phosphate buffer (Sigma) at room temperature for 1 h. The mixture was purified with a desalting column (PD-10 Sephadex column; Cytiva, Marlborough, MA, USA) followed by protein concentration using a 500,000 MW spin column (Vivaspin™; Cytiva) and resuspended in PBS at 500 μg/mL. The quality of anti-FAP or anti-PDPN antibody-IR700 (FAP-IR700 or PDPN-IR700) was confirmed with UV-Vis (Agilent Technologies, Santa Clara, CA, USA), where absorption of the elute was measured at a wavelength of 280 and 689 nm. The antibody-to-IR700 dye ratio was 3:1 for FAP-IR700 and 4:1 for PDPN-IR700. 

### 2.3. In Vitro NIR-PIT

NIH3T3 cells (3 × 10^5^ cells) were seeded into 12-well plates and cultured with TGF-β (20 ng/mL; Peprotech, Cranbury, NJ, USA) in complete media. After 48 h, cells were washed and incubated with or without antibody conjugate (FAP-IR700 or PDPN-IR700) at 20 μg/mL in phosphate-buffered saline (PBS) and incubated for 1 h at 37 °C. Cells were then washed with PBS, and NIR irradiation was performed (150 mW/cm^2^; ML7710 Laser System, Modulight, Tampere, Finland). Cells were cultured for 1 h after PIT and then gently detached from the plates using PBS with 1 mM ethylenediaminetetraacetic acid (EDTA) and a cell scraper for flow cytometry analysis of NIR-PIT efficacy.

### 2.4. Mice

All animal procedures, including housing, were performed in accordance with NIH guidelines and were approved by the institutional Animal Care and Use Committee. Wild-type C57BL/6J mice (strain #000664), as well as Ly5.1 congenic mice (B6.SJL-Ptprc^a^ Pepc^b^/BoyJ; strain # 002014), B6 MMTV-PyVT mice expressing the polyoma virus middle T oncoprotein (PyMT) under the mouse mammary tumor virus (MMTV) promoter (B6.FVB-Tg(MMTV-PyVT)634Mul/LellJ; strain #022974), FAP-TK transgenic mice (B6.Cg-Tg(Fap-TK)MRkl/J; strain #034655) [43], IFN-γ-enhanced yellow fluorescent protein (eYFP) reporter GREAT mice (C.129S4(B6)-Ifngtm3.1Lky/J; strain #017580) [44], and green fluorescent protein (GFP) transgenic mice (C57BL/6-Tg(CAG-EGFP)131Osb/LeySopJ; strain #006567), all on a C57BL/6 background, were purchased from The Jackson Laboratory and maintained in our facility. 

### 2.5. Mouse Tumor Models

Subcutaneous tumors were generated by inoculating 3 × 10^5^ LL/2, MOC2, EL-4, EO771, 4T1, PAN02, or MC38 cells in 100 μL PBS subcutaneously into the right dorsum of mice. Tumor size was measured using electronic calipers (Mitutoyo, Kawasaki, Japan), and tumor volume (V) was calculated as V = (major axis) × (minor axis)^2^ × ½ and followed until endpoint of V = 4000 mm^3^. Mice with a tumor size of approximately 100 mm^3^ were randomly grouped for subsequent experiments. In the B6 MMTV-PyMT mouse, expression of the PyMT oncoprotein is restricted to the mammary epithelium, which results in the appearance of mammary tumors starting from 6 to 8 weeks after birth and pulmonary metastases at 18 weeks [45,46].

### 2.6. In Vivo NIR-PIT

To evaluate the efficacy of FAP-targeted NIR-PIT, mice bearing tumors of approximately 100 mm^3^ were randomized into 4 groups as follows: (1) no treatment (untreated control); (2) 50 μg of anti-FAP antibody intravenous injection, without NIR laser-light exposure (antibody alone); (3) 50 μg of anti-rat IgG1-IR700 antibody intravenous injection with NIR laser-light exposure (isotype control); or (4) 50 μg of anti-FAP-IR700 antibody intravenous injection with NIR laser-light exposure (anti-FAP NIR-PIT). Anti-FAP-IR700, unconjugated anti-FAP, or anti-rat IgG1-IR700 antibody was administered when tumors reached 100 mm^3^ (Day 0). NIR laser-light (690 nm, 150 mW/cm^2^, 50 J/cm^2^, 333 s) exposure of the tumor occurred 24 h later (Day 1). During NIR laser-light delivery, only the tumor was irradiated, and non-irradiated parts of the mouse body were covered with aluminum foil to prevent extraneous exposure. In some experiments, the effect of anti-PDPN NIR-PIT was evaluated using anti-PDPN-IR700 in a similar manner.

### 2.7. Bone Marrow Chimeras

Bone marrow chimera mice were generated by whole-body lethal irradiation (9.5 Gy) of recipient mice using a Cesium-137 irradiator (GammaCell 40, Atomic Energy of Canada Limited, Chalk River, ON, Canada) followed by intravenous injection of donor bone marrow cells within 3 h of irradiation. Bone marrow was collected from donor mice immediately after euthanasia by flushing the epiphysis of cut femurs and tibias with sterile PBS. MMTV-PyVT (expressing Ly5.2) recipients received bone marrow from either FAP-TK (expressing Ly5.2) mice (FAP-TK → MMTV) or Ly5.1 mice (Ly5.1 → MMTV). FAP-TK and Ly5.1 recipients received bone marrow from Ly5.1 (Ly5.1 → FAP-TK) and FAP-TK (FAP-TK → Ly5.1) mice, respectively. Six weeks later, chimerism developed and was examined using peripheral blood by identifying the recipient- and donor-derived cells by Ly5.2 and Ly5.1 markers using flow cytometry where applicable.

### 2.8. In Vivo Depletion of FAP-TK+ Cells by Ganciclovir

FAP-TK mice or bone marrow chimera mice generated using FAP-TK either as recipients or donors underwent depletion of FAP-expressing cells by intraperitoneal administration of ganciclovir (GCV; Sagent Pharmaceuticals, Schaumburg, IL, USA) every 12 h at 100 mg/kg of body weight (2.5 mg per 25 g mouse) in PBS for 2 days (total of 4 doses). 

### 2.9. Flow Cytometry

Anti-FAP and anti-PDPN antibodies (200 µg) were conjugated with phycoerythrin (PE) and allophycocyanin (APC) using R-Phycoerythrin and Allophycocyanin Labeling Kits, respectively (Dojindo Labs, Kumamoto, Japan), according to the manufacturer’s instructions. For evaluation of in vitro PIT, cells were stained with either anti-FAP-PE or anti-PDPN-PE and analyzed using a CytoFLEX (Beckman Coulter, Brea, CA, USA). Tumors were harvested, minced using scissors, and digested using 5 µg/mL collagenase (Liberase TM™, Sigma) in 500 µL complete media for 30 min at 37 °C. Digestion was stopped by the addition of FCS to neutralize protease activity. Digested tissues were then filtered through a 70 μm-pore strainer (BD Biosciences, Franklin Lakes, NJ, USA), centrifuged, washed, and incubated with anti-CD16 antibody (Thermo Fisher Scientific) for blocking Fc. LiveDead™ Fixable Stain (Thermo Fisher Scientific) was used for dead cell exclusion for 10 min at 4 °C. Single-cell suspensions were stained with fluorochrome-conjugated antibodies and analyzed using flow cytometry (CytoFLEX Beckman Coulter). Antibodies and secondary reagents were titrated to determine optimal concentrations. BD™ CompBeads were used for single-color compensation to create multi-color compensation matrices. Data were analyzed using FlowJo 10.8 software (BD Biosciences). See Supplementary Table S1 for detailed information on the antibodies used for flow cytometry.

### 2.10. Histologic and Multiplex Immunofluorescence Staining

Whole tumors (approximately 100 mm^3^) and MMTV-PyMT mouse lung tissue (at the endpoint of mammary tumor growth) were harvested immediately following euthanasia and placed in 10% neutral buffered formalin (NBF) for 24–48 h, followed by 70% ethanol for paraffin embedding. Formalin-fixed, paraffin-embedded (FFPE) sections of 3–5 µm thickness were baked for 30 min at 60 °C and processed for immunohistochemical (IHC) and immunofluorescent (IF) staining. Opal™ Fluorescent Automation IHC Kits (Akoya Bioscience) were used on FFPE tissue according to the manufacturer’s instructions. FFPE tissue slides were stained using a Leica Bond RX autostainer and coverslipped using ProLong Diamond Antifade Mountant (Thermo Fisher Scientific). Hematoxylin and eosin (H&E) staining of FFPE slides was performed by Histoserv, Inc. (Germantown, MD, USA) and American Histolabs, Inc. (Gaithersburg, MD, USA). Antibodies used for histology can be found in Supplementary Table S2. 

### 2.11. Digital Pathology Image Analysis

IHC and IF slides were scanned using a Zeiss AxioScan Z1 whole slide scanner. Multiplex stained slides were scanned in their entirety using a 20× objective lens. Digital image analysis was performed using HALO^®^ Imaging Analysis platform v3.5 (Indica Labs, Albuquerque, NM, USA). A HALO^®^ random forest classifier was used to train a classifier to segment glass, muscle, epithelial, stromal, or necrotic tumor regions on H&E-stained whole tumor digital slides (LL/2 and MMTV-PyVT). Specifically, the classifier was trained based on 5–10 manual annotations of these regions. The classifier was visually and iteratively improved to prevent any inaccurate classification by manually adding additional training examples as needed. A HALO^®^ AI DenseNet v2 classifier was trained to detect metastasis in lung tissue. Whole slide images taken every 20 μm of the entire lung were first annotated to exclude non-lung tissue (esophagus, thyroid, bronchial lymph nodes). Subsequent classified regions were validated by two board-certified veterinary pathologists in consensus. The percentage of each measure (tumor-to-lung ratio) was determined by dividing the total area classified as tumor by total area classified as lung. 

The HALO^®^ High-Plex FL algorithm was used to analyze fluorescent cells and colocalization of markers. Thresholds of each stain were set using the real-time tuning window. User-defined cell phenotypes were created to make the algorithm quantify single-, double-, or triple-positive cells. Cell segmentation was performed with the help of multiple parameters, including minimum nuclear intensity, nuclear contrast threshold, and nuclear and membrane segmentation aggressiveness. Cell phenotype used to create heat maps was defined based on the antigen expressions as the following: DAPI^+^FAP^+^ = FAP^+^ cell, DAPI^+^PDPN^+^ = PDPN^+^ cell, DAPI^+^CD8^+^ = CD8^+^ T cell. To assess the validity of the unsupervised cell phenotyping algorithm, random areas were selected for manual visual counting of positive cell numbers. 

### 2.12. Statistical Analysis

Graphing and statistical analyses were carried out using GraphPad Prism 9 (GraphPad Software, La Jolla, CA, USA). *p*-values were calculated using two-tailed Student’s *t*-test when comparing two experimental groups or one-way ANOVA when comparing more than two experimental groups. Parametric or non-parametric tests were applied accordingly. Error bars represent standard error of the mean unless otherwise noted. 

Figures and schematics were created using BioRender (Biorender.com (accessed on 28 September 2023)).

## 3. Results

### 3.1. CAFs Are Present in the TME of Multiple Tumor Models

**Figure 1:**
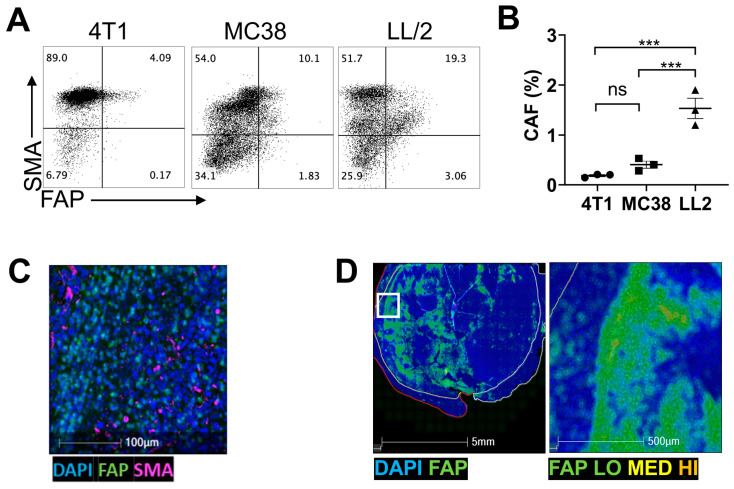
CAFs are present in the TME across several tumor models. (**A**) Representative flow cytometry plots of 3 replicates comparing expression of CAF subset marker SMA and FAP in 4T1, MC38, and LL/2 tumors. (**B**) Frequency of SMA^+^FAP^+^ CAFs analyzed in (**A**) demonstrates significantly higher CAF frequency in LL/2 tumors than in the others. ***: *p* < 0.001; ns: not significant by one-way ANOVA. (**C**) Representative immunofluorescent histology image of three LL/2 tumors expressing activated fibroblast markers α-SMA (pink) and FAP (green). Nuclei were counterstained with DAPI (blue). *n* = 3. (**D**) Heat map of FAP expression generated based on the data of the tumor presented in (**C**). Tumor mass is represented by white line, stromal boundary is represented by yellow line, and tumor invasive front is represented by red line. The area in the white box (left) is magnified in the right panel.

We first examined the expression of five commonly reported CAF markers [7], namely FAP, α-SMA, PDPN, platelet-derived growth factor receptor-alpha (PDGFR-α), and platelet-derived growth factor receptor-beta (PDGFR-β), in seven murine malignant cell lines, EL-4, MC38, EO771, PAN02, LL/2, MOC2, and 4T1 in vitro (Figure S1A,B). All cell lines highly expressed α-SMA, FAP, and PDPN, whereas PDGFR-α expression was low to absent. PDGFR-β was highly expressed by EO771 and PAN02 tumor cells but not by others. Among these cell lines, LL/2, 4T1, and MC38 demonstrated the most reliable growth kinetics in mice. To optimize downstream experiments, these three murine tumor models were compared for the presence of the immunosuppressive CD45^−^α-SMA^+^FAP^+^ CAF subtype [47,48]. Flow cytometry analysis indicated the highest frequency of the CD45^−^α-SMA^+^FAP^+^ cells was in the LL/2 tumors compared with 4T1 and MC38 tumors (Figure 1A,B). Immunofluorescent histological analysis of the LL/2 tumors confirmed the presence of α-SMA^+^ and FAP^+^ cells (Figure 1C), and a heat map analysis showed that FAP^+^ cells were concentrated predominantly at the stromal margin near the invasive front of the tumor (Figure 1D). 

Because the growth of subcutaneously inoculated tumors is more rapid than naturally occurring cancers, and this could affect CAF development and distribution, we performed a similar expression analysis on a genetically engineered mouse model (GEMM) of spontaneous mammary cancer, the MMTV-PyVT mouse [45,46]. Mammary tumors in these GEMMs have similar characteristics to human breast carcinoma, including tumor progression [49] and the presence of CAF subsets [50]. Histologically, the MMTV-PyVT tumors also had a high prevalence of α-SMA^+^FAP^+^ cells at the tumor periphery (Figure S2A). We also found that these cells demonstrated Ki67^+^ staining, indicating their active proliferation (Figure S2B).

### 3.2. Anti-FAP-IR700 NIR-PIT Induces Cell Death of FAP-Expressing Fibroblasts In Vitro

**Figure 2:**
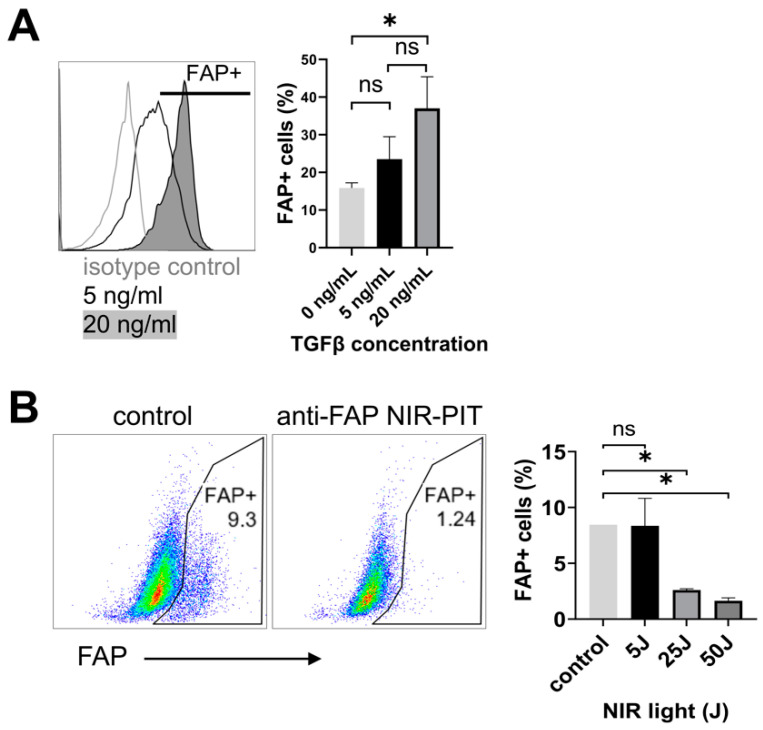
Anti-FAP NIR-PIT induces cell death of FAP^+^-expressing fibroblasts in vitro. (**A**) Representative flow cytometry data of NIH3T3 cells stimulated with 5 or 20 ng/mL of TGF-β indicated induction of FAP in live cells (left, *n* = 3). Cumulative data on frequency of FAP^+^ cells in live cells are shown on the right. *: *p* < 0.05; ns: not significant by one-way ANOVA. (**B**) NIH-3T3 cells stimulated with TGF-β (20 ng/mL) underwent anti-FAP NIR-PIT in vitro. Representative flow cytometry data show the frequency of FAP^+^ cells in live cells 1 hr after NIR-PIT or without treatment (*n* = 3–4 per group). Cumulative data are shown on the right. *: *p* < 0.05; ns: not significant by one-way ANOVA.

To test the efficacy of anti-FAP NIR-PIT, we next performed depletion of FAP^+^ cells in vitro. Murine fibroblasts (NIH3T3) were stimulated with TGF-β to induce FAP expression [51,52]. NIH3T3 cells upregulated FAP^+^ in a TGF-β dose-dependent manner observed via flow cytometry analysis (Figure 2A). In vitro anti-FAP NIR-PIT targeting of FAP^+^ NIH3T3 cells resulted in cell killing that was dependent on NIR light intensity (Figure 2B), confirming the efficacy of anti-FAP NIR-PIT in vitro. Based on these results, we chose 50 J of NIR light exposure for subsequent in vivo PIT experiments. 

### 3.3. Anti-FAP NIR-PIT Suppresses Tumor Growth and Lung Metastasis In Vivo

**Figure 3:**
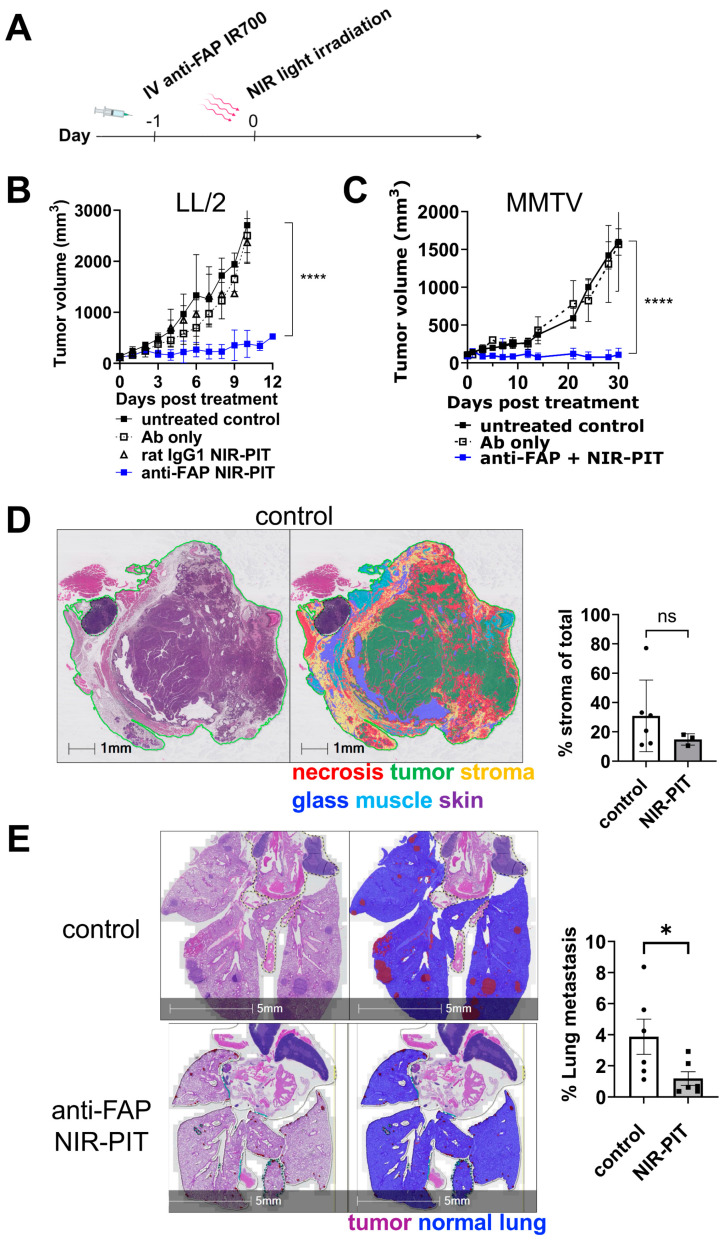
Anti-FAP NIR-PIT suppresses tumor growth and lung metastases in vivo. (**A**) Experimental scheme for in vivo NIR-PIT experiments. (**B**) Groups of mice bearing LL/2 tumors received an intravenous injection of either unconjugated anti-FAP (Ab only), rat IgG1-IR700 (isotype control), or anti-FAP IR700 (anti-FAP NIR-PIT) antibodies at Day 1, and the tumors in both isotype control and anti-FAP groups were exposed to 50 J NIR light at 24 h post-antibody injection (Day 0). The average tumor volume of the groups, including that of untreated group, was plotted (*n* = 5–6 per group). ****: *p* < 0.0001; ns: not significant by one-way ANOVA. (**C**) MMTV-PyVT mice received similar treatments against one of the spontaneously grown mammary tumors, and the volume of the tumors was plotted (*n* = 5–6 per group). ****: *p* < 0.0001; ns: not significant by one-way ANOVA. (**D**) Representative H&E-stained histology section with analysis markup from random forest-classified tissue regions for an untreated MMTV-PyVT mammary tumor. The percentage of stroma area in total tumor area, including necrosis, in the control (*n* = 6) and anti-FAP NIR-PIT treatment (*n* = 3) groups is summarized in the graph. ns: not significant by two-tailed Student’s *t*-test. (**E**) H&E sections of lungs of MMTV-PyVT mice with endpoint mammary tumors (**left panels**) were AI-classified for lung metastasis (**right panels**). Representative data of control (**top panels**) and anti-FAP NIR-PIT treatment groups (**bottom panels**) are shown. *n* = 6 per group. *: *p* < 0.05 by two-tailed Student’s *t*-test.

In vivo anti-FAP NIR-PIT was performed in subcutaneous LL/2, and spontaneously arising MMTV-PyVT mammary tumors to evaluate therapeutic efficacy on tumor growth. In both models, tumor-bearing mice were given anti-FAP-IR700 conjugate on Day −1, NIR-PIT was then performed 24 h later (Day 0), and tumors were measured for 10–30 days (Figure 3A). Anti-FAP NIR-PIT significantly suppressed LL/2 tumor growth compared with anti-FAP Ab alone, rat IgG1 isotype control plus NIR-PIT light exposure, or an untreated control group (Figure 3B). Similarly, MMTV-PyVT tumor growth was significantly suppressed in the anti-FAP NIR-PIT group compared with anti-FAP Ab alone or an untreated control group (Figure 3C). Digital image analysis to segment tumor regions (as tumor, stroma, necrosis, muscle, skin, or slide glass) was performed on H&E-stained scanned sections of MMTV-PyVT untreated control and anti-FAP NIR-PIT treatment groups using a random forest classifier. The average stromal area (total stromal area divided by total classified area) was reduced by approximately 50% at 24 h post NIR-PIT compared to untreated controls, although this change was not significant (Figure 3D). Moreover, in contrast to the multiple lung metastases observed in the mice with untreated MMTV-PyVT tumors, lung metastases were significantly reduced in the anti-FAP NIR-PIT group (Figure 3E).

### 3.4. Anti-PDPN-IR700 NIR-PIT Induces Cell Death of PDPN-Expressing Fibroblasts In Vitro but Did Not Suppress Tumor Growth In Vivo

In addition to FAP, a second CAF marker, podoplanin (PDPN), was tested for efficacy as an anti-CAF NIR-PIT target. As with FAP, PDPN expression increased in NIH3T3 cells in vitro after stimulation with TGF-β (Figure S3A). Anti-PDPN NIR-PIT demonstrated killing of NIH3T3 cells compared to untreated control cells (Figure S3B). PDPN expression levels varied among tumor types in vivo (Figure S3C), with the highest expression in MOC2 tumors compared to LL/2 and MC38 tumors but absent in MOC2 in vitro-cultured cells. PDPN-expressing cells were observed predominantly at the tumor periphery, near the invasive front (Figure S3D). Anti-PDPN NIR-PIT was performed on mice inoculated with subcutaneous MOC2 tumors; however, no difference in tumor growth was observed between the anti-PDPN NIR-PIT group, anti-PDPN Ab alone, or an untreated control group (Figure S3E). 

### 3.5. Anti-FAP NIR-PIT Increases Immune Effector Cells and Their IFN-γ Expression in the Tumor Microenvironment

**Figure 4:**
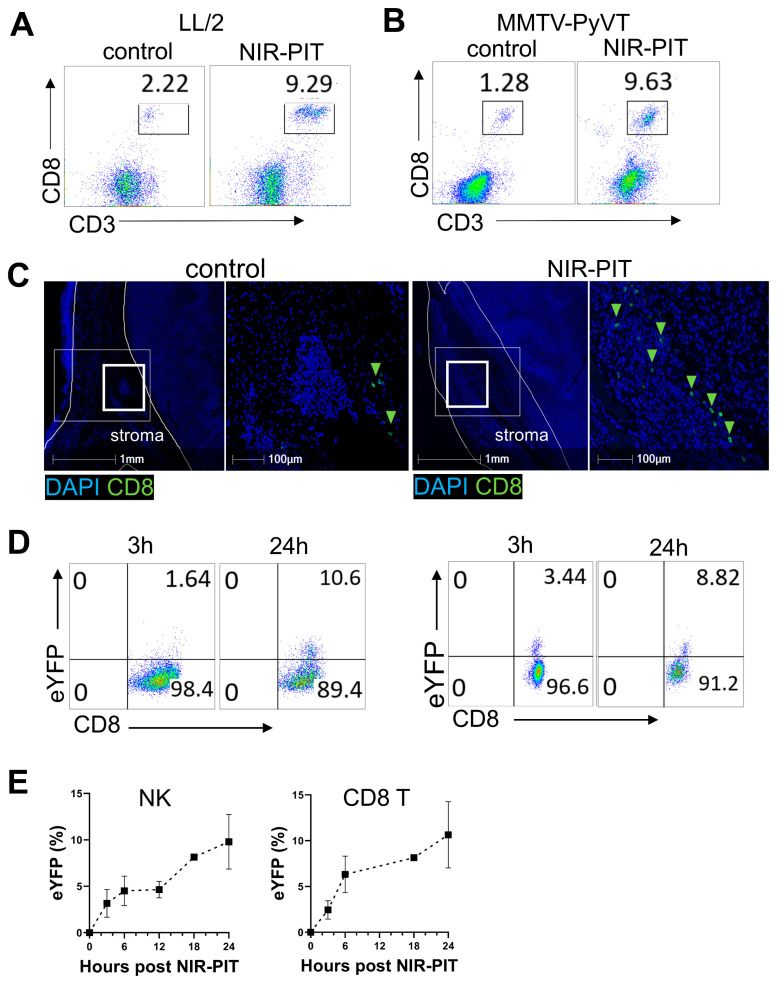
Anti-FAP NIR-PIT increases immune effector cells and IFN-γ in the TME. (**A**) Flow cytometry analysis of CD3^+^CD8^+^ T cell frequency in live CD45^+^ cells collected from the LL/2 tumor, untreated or 24 h after anti-FAP NIR-PIT (representative data of *n* = 3). (**B**) Flow cytometry analysis of CD3^+^CD8^+^ T cell frequency in live CD45^+^ cells found in an MMTV-PyVT tumor with and without anti-FAP NIR-PIT (representative data of *n* = 3). (**C**) Multiplex immunohistochemistry of CD8 T cells in control (**left 2 panels**) or 24 h after anti-FAP NIR-PIT (**right 2 panels**) LL/2 tumor (blue: DAPI; green: CD8). In each condition, the area in the bold white box in the left is magnified in the right panel. Representative images of *n* = 3–5. (**D**) Representative flow cytometry data of CD8 T cells collected from LL/2 tumors in eYFP-IFN-γ reporter GREAT mice at 3 h and 24 h post-anti-FAP NIR-PIT. Fractions of CD8 T cells and NK cells were eYFP positive, indicating IFN-γ production. (**E**) The fraction of IFN-γ producing (eYFP positive) CD3^-^NK1.1^+^ cells and CD3^+^CD8^+^ T cells from 0 h to 24 h after anti-FAP NIR-PIT against LL/2 tumors in GREAT mice were plotted (*n* = 3–5 per time point).

To understand the mechanism of tumor growth suppression, the frequency of key anti-tumor effector cells, such as cytotoxic CD8 T and natural killer (NK) cells, as well as their IFN-γ production, was measured within the tumor. As the primary anti-tumor effector cells, CD8 T and NK cells were enumerated in the tumor before and 24 h after anti-FAP NIR-PIT. Flow cytometry showed an increase in the frequency of CD8 T cells after anti-FAP NIR-PIT for both LL/2 (Figure 4A) and MMTV-PyVT tumor models (Figure 4B). Multiplex immunohistochemistry revealed that stromal distribution of CD8 T cells 24 h after anti-FAP NIR-PIT was increased compared to untreated controls (Figure 4C). To examine IFN-γ production in effector cells, GREAT eYFP-IFN-γ reporter mice were inoculated with LL/2 tumors and analyzed for the induction of eYFP signal in intratumoral CD8 T and NK cells by flow cytometry. As early as 3 h after treatment, CD8 T cells and NK cells began to express eYFP, indicating the production of IFN-γ (Figure 4D). eYFP positivity in CD8 T and NK cells increased from 3 h to 24 h (Figure 4D,E), suggesting IFN-γ production continued at least up to 24 h after anti-FAP NIR-PIT. 

### 3.6. Depletion of FAP^+^ CAFs and FAP^+^ Hematopoietic Cells Both Contribute to Tumor Suppression in Anti-FAP NIR-PIT

**Figure 5:**
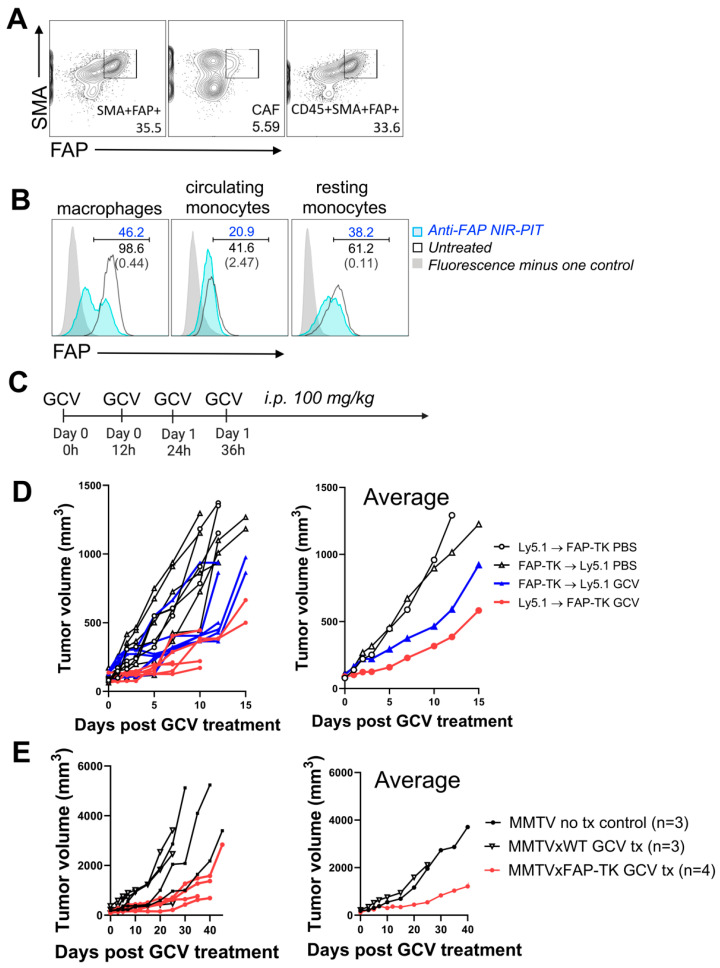
Depletion of FAP^+^ stromal cells, as well as that of hematopoietic cells, contributes to tumor growth suppression. (**A**) Flow cytometry analysis of FAP^+^SMA^+^ cell frequency in live GFP+ cells (endogenous cells, **left**), live GFP^+^CD45^-^ cells (CAFs, **middle**), and live GFP^+^CD45^+^FAP^+^SMA^+^ cells (leukocyte, **right**) in LL/2 tumor grown in GFP-Tg mice. Representative data of *n* = 4. (**B**) Flow cytometry analysis of FAP expression in live CD45^+^CD11b^+^F4/80^high^ macrophages, live CD45^+^CD11b^+^Ly6C^high^ circulating monocytes, and live CD45^+^CD11b^+^Ly6C^int^ resting monocytes in LL/2 tumor, untreated (black line) and 1 h after anti-FAP NIR-PIT (blue line). Representative data of *n* = 2. (**C**) Experimental timeline for FAP^+^ cell depletion by 100 mg/kg ganciclovir (GCV) intraperitoneal administration in FAP-TK mice or bone marrow chimera mice generated using FAP-TK mice bearing LL/2 tumor. (**D**) Growth curves of LL/2 tumors in bone marrow chimera experimental groups following GCV administration. Growth of each tumor (**left**) and the average (**right**) are shown. *n* = 7 for FAP-TK → Ly5.1 GCV group, *n* = 5 for FAP-TK → Ly5.1 PBS group, *n* = 6 for FAP-TK → Ly5.1 GCV group, *n* = 4 for Ly5.1 → FAP-TK PBS group. (**E**) Growth curves of LL/2 tumor in MMTV-PyVT bone marrow chimera experimental groups following GCV administration. Growth of each tumor (**left**) and the average (**right**) are shown. *n* = 3 for MMTV-PyVT no treatment control group (MMTV no tx control), *n* = 3 for Ly5.1 → MMTV GCV group, and *n* = 4 for FAP-TK → MMTV GCV group.

Flow cytometry characterization of the FAP^+^ population in GFP mice inoculated with LL/2 tumors demonstrated that many endogenous GFP^+^FAP^+^ cells also expressed CD45 (Figure 5A), a pan-leukocyte marker. Further analysis of this FAP^+^CD45^+^ population in anti-FAP NIR-PIT treated and untreated LL/2 tumors suggested that FAP^+^ macrophages (CD45^+^Ly6C^int^F4/80^high^), FAP^+^-circulating monocytes (CD45^+^Ly6C^high^F4/80^int^), and FAP^+^ resting monocytes (CD45^+^Ly6C^low^F4/80^int^) were depleted within 1 h by anti-FAP NIR-PIT (Figure 5B; int: intermediate). 

To determine whether the tumor suppressive effect observed with anti-FAP NIR-PIT was due to ablation of FAP^+^CD45^-^ CAFs or that of FAP^+^CD45^+^ hematopoietic cells, a series of FAP-TK bone marrow chimeras were generated to allow for selective FAP^+^ cell depletion of either stromal or hematopoietic cells. Reconstitution of PBMC with transferred bone marrow-derived cells was confirmed to be >95% by flow cytometry analysis using Ly5.1 and Ly5.2 congenic markers (Figure S4A). Generated bone marrow chimeras and control FAP-TK or wild-type mice received four doses of 100 mg/kg GCV via intraperitoneal injections every 12 h (Figure 5C). GCV treatment depletes actively dividing thymidine kinase (TK)-expressing cells, which, in our study, are FAP-TK cells. Depletion of FAP^+^ cells in LL/2 tumors in FAP-TK mice was confirmed by flow cytometry (Figure S4B). In the chimeras, GCV administration selectively depleted actively dividing FAP^+^ cells in either the stromal (when FAP-TK mice were recipients) or hematopoietic compartments (when bone marrow from FAP-TK mice were transferred as donor cells). Suppression of tumor growth was observed for the LL/2 stromal FAP^+^ cell depletion group (Ly5.1 → FAP-TK) and, to a lesser extent, for the hematopoietic FAP^+^ depletion group (FAP-TK → Ly5.1) compared with PBS injected controls (Figure 5D). Tumor suppression was also observed for the MMTV-PyVT hematopoietic FAP^+^ cell depletion group (FAP-TK → MMTV) compared with a bone marrow transfer control group (Ly5.1 → MMTV) receiving GCV, and an untreated control group, although to a lesser degree compared to that of the stromal FAP^+^ cell depletion group (Figure 5E). These data indicate that both depletion of FAP^+^ CAFs and FAP^+^ hematopoietic cells can suppress the growth of cancers, and therefore, anti-FAP NIR-PIT depletes both the FAP^+^ CAFs and FAP^+^ hematopoietic cells (Figure 5B), demonstrating that both cell types are important to the efficacy of FAP-targeted NIR-PIT.

## 4. Discussion

Cancer-associated fibroblasts (CAFs) comprise a majority of the stromal cellular compartment in solid tumors and serve significant functions in immunosuppression, invasion, and angiogenesis. One of the greatest challenges in targeting CAFs is the lack of a pan-specific biomarker, as CAFs often express variable phenotypes across tumors and tissue types. High expression of FAP by CAFs is a negative prognostic indicator thought to be due to their immunosuppressive properties, but FAP^+^ cells are a promising intratumoral target for the depletion of selected stromal cells [4,5,6]. FAP itself also directly supports tumor growth, invasion, and metastasis through extracellular matrix remodeling [8]. Consistent with previous reports, we observed the expression of FAP in several murine cancer models and chose it as a CAF target for NIR-PIT. An additional marker, PDPN, was another target marker for comparison. In selecting tumor models for our study, we used immune-competent tumor models, including a GEMM, to better reflect relevant cancer biology and CAF ecology. While co-transplantation of human stromal cells (fibroblasts) with cancer cells has been previously performed, the resulting phenotype is difficult to reproduce and does not reflect the natural distribution of CAFs [53]. Instead, we chose one syngeneic subcutaneous lung cancer LL/2 and one spontaneously occurring mammary cancer with frequent lung metastasis (MMTV-PyVT GEMM) and investigated the therapeutic and immune effects of FAP-targeted NIR-PIT. To our knowledge, this work is the first to use anti-FAP NIR-PIT to deplete CAFs in the TME as well as in spontaneously developed MMTV-PyVT mammary tumors, which more closely mimic the human TME, including cellular and spatial heterogeneity, tumor growth, and CAF subsets [49]. The KPC (Kras^G12D/+^; Trp53^R172H/+^; P48-Cre) mouse, a spontaneous model of pancreatic ductal adenocarcinoma, could also be considered for future work examining the effects of FAP^+^ cell depletion, as it is a highly stromagenic cancer [54]. 

Previous studies have indicated that anti-FAP therapies, such as blocking FAP-α or inhibiting the enzymatic activity of FAP-α, are safe and effective in preclinical studies but have not proven effective in human clinical trials [34,55]. NIR-PIT targeting EGFR-expressing cancer cells has been approved for clinical use in Japan (Rakuten Medical Inc.) and is currently in Phase 3 clinical trials in the United States (ClinicalTrials.gov Identifier: NCT02422979) [38], indicating a promising level of safety and efficacy in humans. NIR-PIT targeting CD25 was also used to deplete immunosuppressive Treg cells within the tumor [39,56], while NIR-PIT targeting FAP has been used to deplete in vitro stimulated CAFs [40,42,57] from the TME. As NIR-PIT selectively kills the cells bound with the antibody-IR700 conjugate without affecting the neighboring cells [37] and NIR light is carefully applied only at the tumor site, off-target effects are minimal [39,56], and local, transient edema observed at the NIR light irradiation site resolved within 48 h. We demonstrate that local, anti-FAP NIR-PIT can successfully and selectively deplete FAP^+^ immunosuppressive CAFs and myeloid cells in the TME, which results in activation of CD8 T and NK cells, production of IFN-γ, and suppression of tumor growth in both the subcutaneous LL/2 and spontaneous MMTV-PyVT tumors. CAFs suppress CD8 T cell cytotoxic activity and recruitment in TME, in part through chemokines/cytokines such as TGF-β and CXCL12, and immunosuppressive M2 macrophages exert T cell inhibition via TGF-β and IL-10 [58]. CAFs also suppress the cytotoxic function of NK cells through interactions with the inhibitory receptors expressed on the NK cells [58]. Depletion of FAP^+^ CAFs and myeloid cells by anti-FAP NIR-PIT enabled CD8 T cells and NK cells to regain their cytotoxic function and possible influx to the TME. Removal of the physical barrier consisting of CAFs in the periphery of the tumor and decreased intratumoral pressure could also allow increased T cell infiltration into the tumor. Although a single anti-FAP NIR-PIT treatment did not result in complete tumor ablation, lung metastases in MMTV-PyVT tumor mice were significantly reduced. Repeating the NIR light exposure without repeating the antibody-IR700 infusion may increase efficacy [39].

It has been reported that higher PDPN expression is associated with poorer outcomes in human colorectal carcinomas [59] and is considered to be another promising method of targeting CAFs [60]. Aside from high expression on CAFs, PDPN is also highly expressed in lymphatic endothelium, both in normal tissues and during tumorigenesis. Additionally, PDPN serves important functions during development [61,62,63] and may not be an ideal target molecule for systemic depletion. We hypothesized that NIR-PIT could allow selective depletion of PDPN^+^ cells within the TME and examined the efficacy of anti-PDPN NIR-PIT in a syngeneic MOC2 tumor model. Despite being effective in vitro against murine fibroblasts (NIH3T3) with upregulated PDPN expression, anti-PDPN NIR-PIT did not reduce tumor volume in vivo in our study. Possible explanations include ineffective antibody distribution in the tumor or less specificity of the PDPN-IR700 conjugate for CAFs in vivo. Taken together, we conclude that anti-PDPN NIR-PIT was a less favorable strategy for targeting CAFs compared with anti-FAP NIR-PIT. 

There are several potential future clinical applications of anti-CAF-directed NIR-PIT. Anti-FAP NIR-PIT could be considered as an adjunctive therapy with other cancer drugs, such as cytotoxic treatment or immune checkpoint inhibitors. Such combination therapies could potentially enhance the immune response following NIR-PIT and, thus, the therapeutic outcome. In previous cancer cell targeting NIR-PIT, a phenomenon known as the super-enhanced permeability and retention (SUPR) effect was observed [64,65]. Tumors treated with NIR-PIT underwent a rapid period of increased vascular permeability after initial cell killing, resulting in enhanced delivery of nano-sized therapeutic agents. By eliminating fibroblasts with anti-FAP NIR-PIT, we may see a similar phenomenon due to the reduction of CAFs and subsequently increased permeability due to a reduction in tumor pressure. Anti-CAF NIR-PIT could also be considered in the context of other fibrotic conditions. CAFs have been described to contribute to non-malignant disease conditions, including cardiac fibrosis [66] and inflammatory bowel disease [67], and selective depletion of these cells might help us better understand the underlying role of fibroblasts in the mechanism of disease [68]. One area of research with much recent interest is the use of FAP inhibitors (FAPi) as a PET imaging agent in cancer patients whose tumors or TME express FAP more highly than those in mice [69]. Radiolabeled FAPi tracers bind to FAP^+^ cells abundant in cancers and distinguish the tumor from surrounding normal structures in imaging. Accumulating data [70,71,72] suggest FAPi PET imaging [73] is particularly helpful in the detection of peritoneal ovarian, pancreatic, and gastric carcinomatosis [74]. FAPi PET could also be useful in identifying cancer patients who might benefit from anti-FAP therapy. Furthermore, as human tumors express higher levels of FAP [69] than mouse tumors, anti-FAP NIR-PIT could demonstrate even more benefit in the human cancer clinical setting than in preclinical murine studies.

We found that FAP was expressed in CAFs but also in other immune cell types. Our flow cytometry analysis data indicated that these FAP^+^CD45^+^ cells were likely myeloid cells, consistent with previous work demonstrating that macrophages, specifically F4/80^high^/CCR2^+^/CD206^+^ M2 or tumor-associated macrophages (TAMs), express FAP [75]. From our data in bone marrow chimeras using FAP-TK recipients or donors, depletion of FAP^+^ stromal cells and FAP^+^ hematopoietic cells by GCV together had a substantial suppressive tumor effect greater than the effect of either alone. This result supports the hypothesis that the FAP^+^ hematopoietic cell subset likely contributed to the total observed tumor suppression of anti-FAP NIR-PIT in non-chimeric mice. This hypothesis is also consistent with previous observations that ablation of FAP^+^/F4/80^high^ TAMs using a diphtheria toxin receptor (DTR) model results in the suppression of tumor growth [75]. In the intratumoral FAP^+^ cell depletion by anti-FAP NIR-PIT, it is likely that depletion of CAFs and hematopoietic FAP-expressing myeloid cells both contributed to the observed therapeutic effect. Thus, anti-FAP NIR-PIT targets different immunosuppressive cell types in the TME with one treatment.

This study has some limitations. We used GREAT eYFP-IFN-γ reporter mice to analyze IFN-γ production in CD8 T and NK cells by flow cytometry. While this method excludes any ambiguity caused by varying background staining levels often found in IFN-γ intracellular staining, eYFP protein can remain within a cell after IFN-γ is no longer produced, and is therefore difficult to determine the true peak of IFN-γ production. However, the reporter system allowed us to analyze the production of IFN-γ in the TME without ex vivo re-stimulation of the harvested cells and without reduction of IFN-γ detection due to the loss of activation status during the long tumor processing time, which occurs when analyzed without ex vivo re-stimulation. Another limitation is that ganciclovir, used for the depletion of FAP^+^ cells using the FAP-TK/GCV system in the chimera models, may also exhibit generalized cytotoxicity. To achieve FAP depletion in the FAP-TK chimeras, a near-toxic systemic dose (100 mg/kg intraperitoneal injection twice daily for two days) was used. Some studies have reported that systemic depletion of FAP^+^ cells induced bone marrow hypocellularity, anemia, and cachexia in mice [76,77]. With the dose of GCV used in our study, we cannot exclude the possibility of inducing some off-target effects in GCV-treated mice. In addition, GCV induces systemic depletion of FAP^+^ cells, whereas NIR-PIT depletes FAP^+^ cells locally in tumors. The contribution of CD45^+^FAP^+^ cell depletion by NIR-PIT on the therapy efficacy could be smaller than that by GCV-induced depletion.

In NIR-PIT, the NIR light at a wavelength of 690 nm can only penetrate and treat cancers at a maximum depth of approximately 1 cm. NIR light, however, can be delivered through a catheter needle or endoscope, thereby expanding its use in deeper tumors and metastatic lesions. Larger lesions can be treated by an array of multiple needles [78,79]. It is also possible that additional round(s) of the antibody-IR700 conjugate infusion and NIR-PIT could improve treatment efficacy even further. Such repeated NIR-PIT could prevent tumor regrowth, which occurs months or years later in human patients, in contrast to the experimental mouse tumor models [53]. 

## 5. Conclusions

In summary, this work provides evidence to support anti-FAP NIR-PIT as a viable method for the depletion of immunosuppressive CAFs. The selective depletion of FAP^+^ cells using NIR-PIT successfully suppressed tumor growth in a subcutaneous mouse tumor model and lung metastasis in a spontaneous mouse mammary tumor model. Our findings highlight a promising therapeutic approach for selectively and safely eliminating FAP^+^ cells within the tumor microenvironment.

## Data Availability

The data presented in this study are contained within the article or the Supplementary Materials and are also available upon request from the corresponding author.

## Supplementary Materials

The following supporting information can be downloaded at https://www.mdpi.com/article/10.3390/cancers16020449/s1. Table S1: Antibodies used for flow cytometry; Table S2: Antibodies used for histology; Figure S1: Tumor cell expression of CAF markers in vitro; Figure S2: CAFs are present in the TME of MMTV-PyVT tumors; Figure S3: Anti-PDPN NIR-PIT depletes PDPN^+^ cells in vitro but did not suppress tumor growth in vivo; Figure S4: Validation of bone marrow chimera and depletion of FAP^+^ cells in tumor in FAP-TK mice.
