# Supplementary material for: Tumor Suppression by Anti-Fibroblast Activation Protein Near-Infrared Photoimmunotherapy Targeting Cancer-Associated Fibroblasts
Source: Cancers (Basel). 2024 Jan 20;16(2):449. doi: 10.3390/cancers16020449 (PMC10813865; doi:10.3390/cancers16020449)
Supplement: Supplementary file 1 [file cancers-16-00449-s001.zip › cancers-2819873-supplementary.pdf]

Supplementary Materials for

Tumor suppression by anti-fibroblast activation protein near-infrared photoimmunotherapy targeting cancer-associated fibroblasts

Raisa A. Glabman[1,2], Colleen P. Olkowski[1], Hannah A, Minor[1], Laura L. Bassel[3], Noemi Kedei[4], Peter L. Choyke[1] and Noriko Sato[1,*]

[1] Molecular Imaging Branch, Center for Cancer Research, National Cancer Institute, National Institutes of Health, Bethesda, MD 20892, USA

[2] Department of Pathobiology and Diagnostic Investigation, College of Veterinary Medicine, Michigan State University, East Lansing, MI 48824, USA

[3] Center for Advanced Preclinical Research, Frederick National Laboratory for Cancer Research, National Cancer Institute, National Institutes of Health, Frederick, MD 21701, USA

[4] Collaborative Protein Technology Resources, Office of Science and Technology Resources, Center for Cancer Research, National Cancer Institute, National Institutes of Health, Bethesda, MD 20892, USA

* Correspondence: saton@mail.nih.gov; Tel.: +1-240-858-3079

Supplementary Tables

Table S1: Antibodies used for flow cytometry

Reagent	Source	Identifier
Mouse anti-mouse FAP (clone 73.3)	Sigma	MABC1145
Rat anti-mouse FAP (clone 983802)	R&D Systems	AB9727
Mouse anti-mouse α-SMA-PE (clone 1A4)	Novus	NBP2-34522PE
Mouse anti-mouse α-SMA-FITC (clone 1A4)	Sigma	F3777
Armenian Hamster anti-mouse CD3-BV421 (clone 145-2C11)	Biolegend	100336
Anti-mouse CD8a-PECy5 (clone 53-6.7)	Thermo Fisher Scientific	15-0081082
Rat anti-mouse CD11b-PECy7 (clone M1/70)	eBioscience	25-0112-82
Rat anti-mouse polyclonal CD16	Biolegend	101301
Mouse anti-mouse CD45.1-FITC (clone: A20)	Thermo Fisher Scientific	11-0453-85
Mouse anti-mouse CD45.2-PE (clone: 104)	Thermo Fisher Scientific	12-0454-83
Mouse anti-mouse CD45.2-BV 650 (clone: 104)	Biolegend	50-402-986
Rat anti-mouse F4/80-APC	Biolegend	123116
LiveDead Fixable Viability Dye eFlour™ 455 UV	Thermo Fisher Scientific	65-0868-14
Rat anti-mouse Ly6C PE (clone: HK1.4)	Thermo Fisher Scientific	12-5932-82
Syrian Hamster anti-mouse PDPN-PE Cy7 (clone 8.1.1)	Biolegend	25-5381-82
Rat anti-mouse PDGFR-α-Super Bright ™ 600 (clone APA5)	Thermo Fisher Scientific	63-1401-82
Rat anti-mouse PDGFR-β-PE (clone APB5)	Thermo Fisher Scientific	14-1402-81
Mouse anti-mouse NK1.1-PE (clone: PK136)	Thermo Fisher Scientific	12-5941-83

Table S2: Antibodies used for histology

Reagent	Source	Identifier
Rabbit polyclonal anti-mouse FAP	Abcam	218164
Rabbit anti-mouse α-SMA (clone EPR5368)	Abcam	Ab124964
Rabbit polyclonal anti-mouse CD3e	Thermo Fisher Scientific	PA1-29547
Rabbit anti-mouse CD8a (clone D4W2Z)	Cell Signaling	989415
Rat anti-mouse CD8a (clone C8/144B)	Invitrogen	MA5-13473
Rabbit anti-mouse CD31 (clone D8V9E)	Cell Signaling	77699S
Rabbit anti-mouse CD45 (clone D3F8Q)	Cell Signaling	70257S
Rabbit anti-mouse Ki67 (clone SP8)	Cell Marque	275R
Rabbit anti-mouse Podoplanin (clone 66)	Invitrogen	MA5-29742

Supplementary Figures

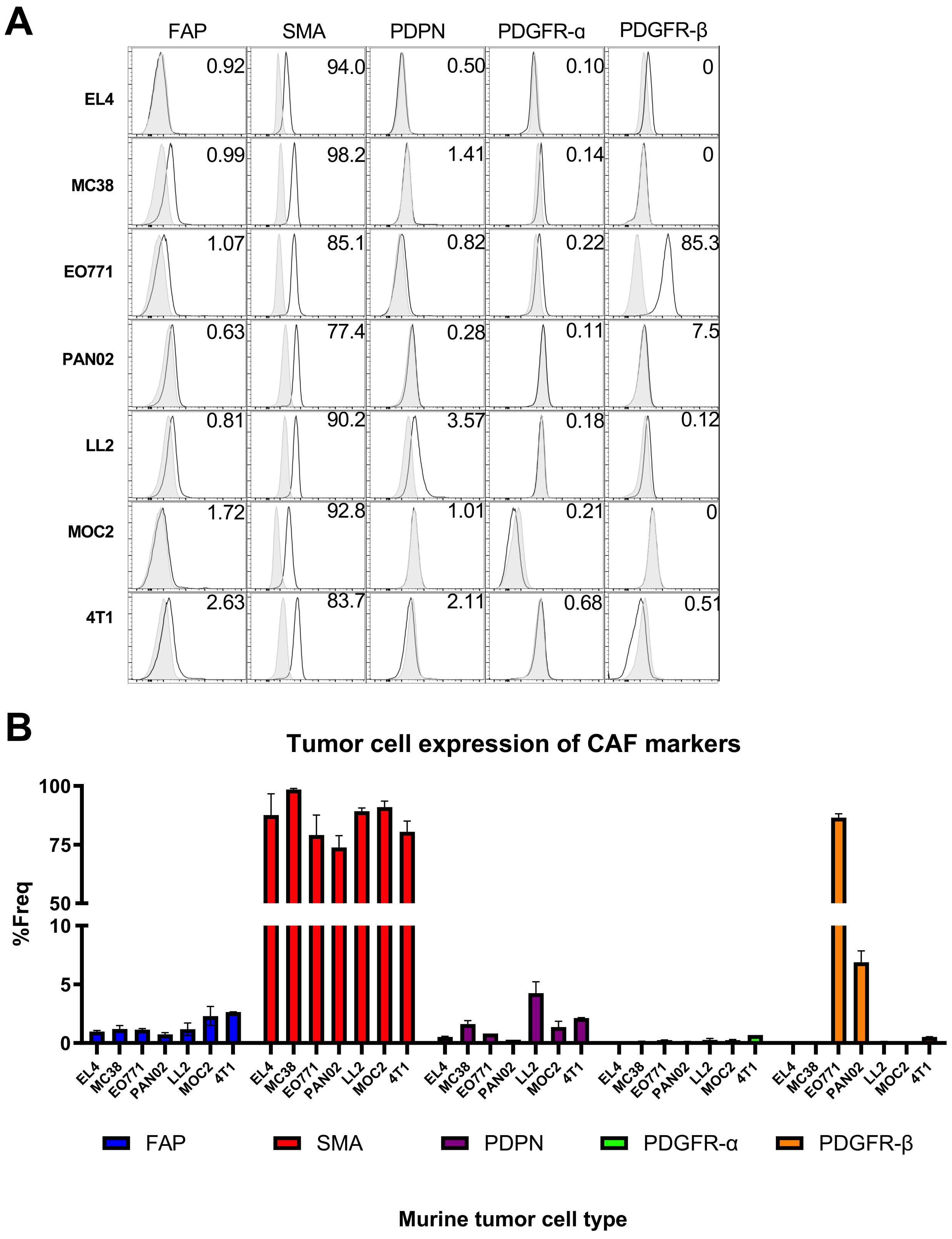

Figure S1: Tumor cell expression of CAF markers in vitro.

(A). Flow cytometry analysis of expression of 5 major CAF markers in murine tumor cell lines expressed as percentage in the Live/Dead staining negative live cells. Representative histograms of 3 replicates showing the specific marker staining (black line) and isotype control staining (gray). The numbers in each panel indicate positive cell frequencies for the CAF marker (top) and the isotype control (bottom) staining. FAP: fibroblast activation protein; SMA: alpha smooth muscle actin, PDPN: Podoplanin, PDGFR-α: platelet derived growth factor receptor alpha; PDGFR-β: platelet derived growth factor receptor beta.

(B). Cumulative data analyzed in A.

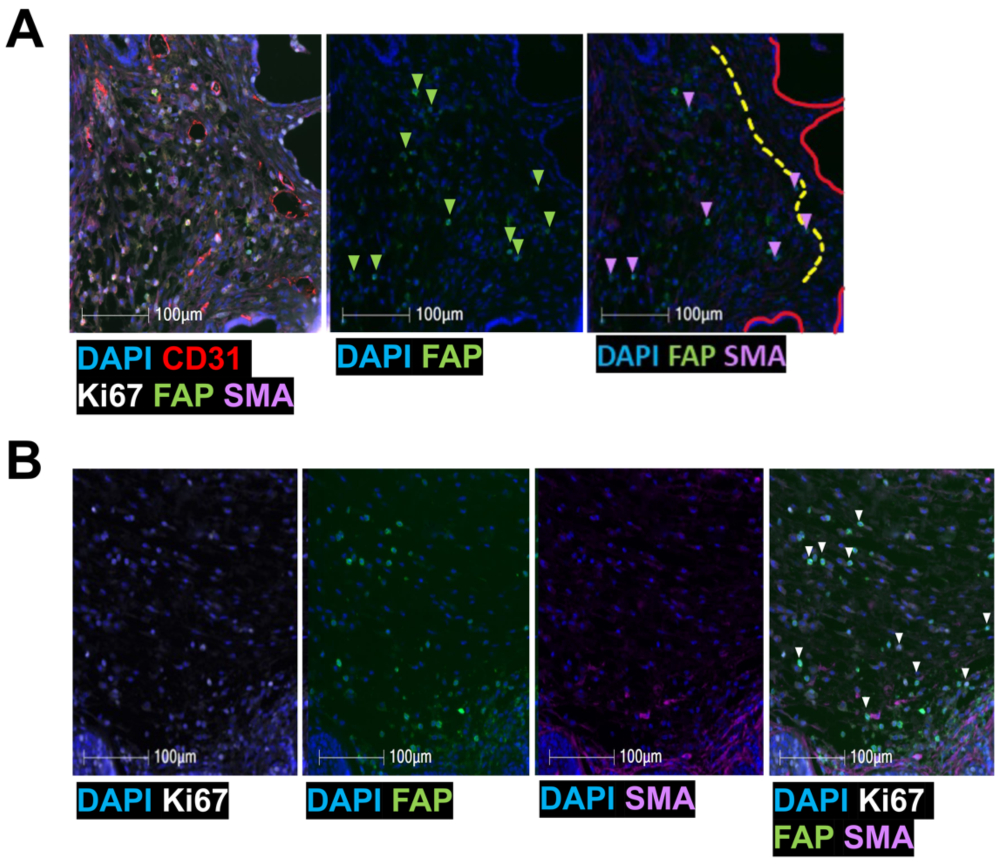

Figure S2: CAFs are present in the TME of MMTV-PyVT tumors.

(A). Immunofluorescent histology of CAF marker expression in the tumors developed in MMTV-PyMT mice. Representative images show expression of the activated fibroblast markers α-SMA (pink) and FAP (green), proliferative marker Ki67 (white), and endothelial marker CD31 (red) with nuclei counterstained with DAPI (blue). Left: merged image, middle: Showing only FAP (green arrow heads) and DAPI staining, right: α-SMA and FAP co-expressing cells (purple arrowheads). Stromal boundary is represented by dashed yellow line and tumor invasive front is shown by solid red line. n=4.

(B). Additional representative images of the MMTV-PyMT tumor. Ki67, FAP and α-SMA staining with DAPI, and the merged image (far right) are presented. White arrowheads indicate FAP+ cells with Ki67 expression. n=3.

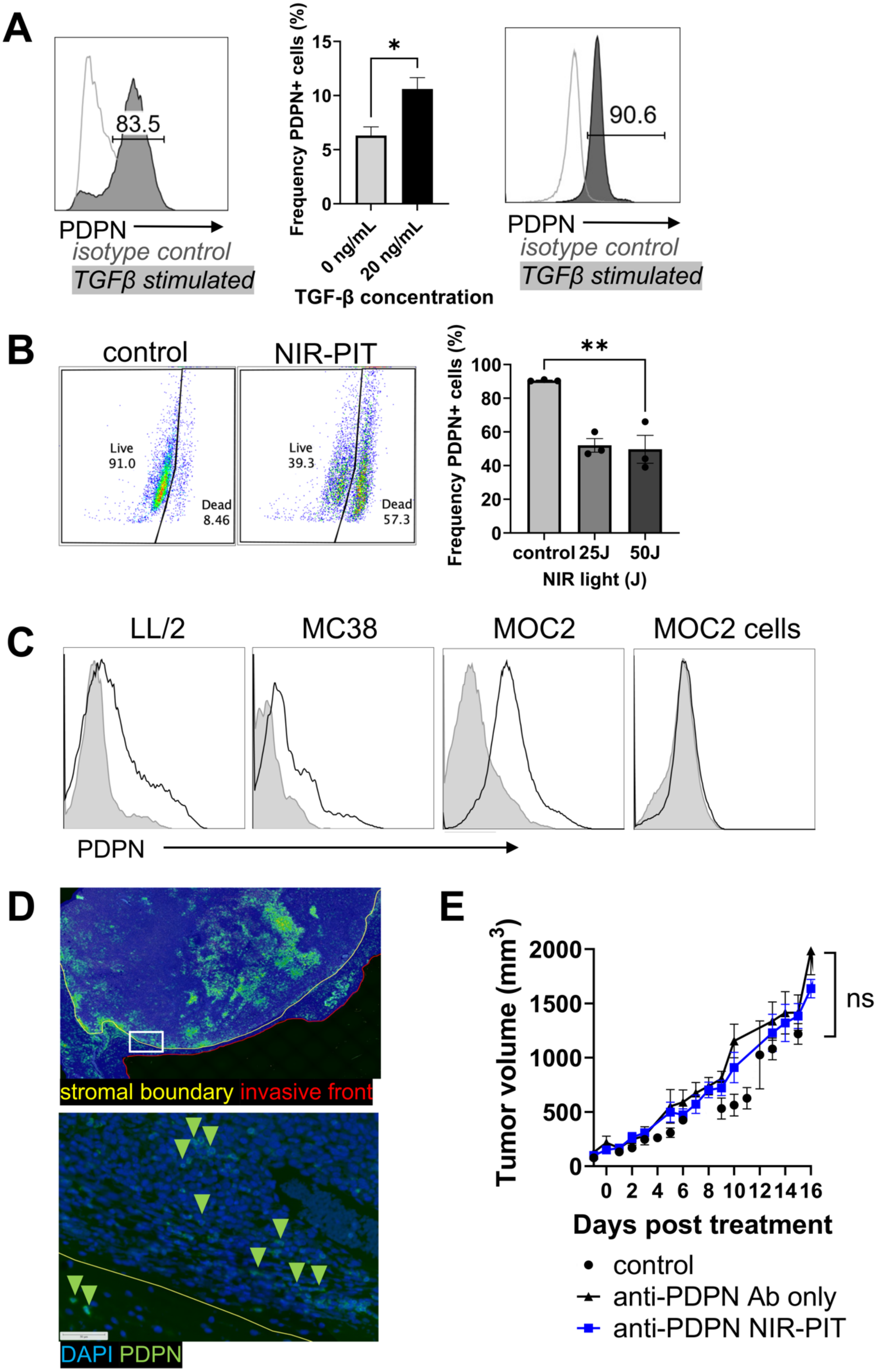

A
83.5
PDPN
isotype control
TGFβ stimulated
Frequency PDPN+ cells (%)
15
10
5
0
*
0 ng/mL
20 ng/mL
TGF-β concentration
90.6
PDPN
isotype control
TGFβ stimulated
B
control
NIR-PIT
Live
91.0
Dead
8.46
Live
39.3
Dead
57.3
Frequency PDPN+ cells (%)
100
80
60
40
20
0
**
control
25J
50J
NIR light (J)
C
LL/2
MC38
MOC2
MOC2 cells
PDPN
D
stromal boundary invasive front
DAPI PDPN
E
Tumor volume (mm3)
2000
1500
1000
500
0
0 2 4 6 8 10 12 14 16
Days post treatment
ns
control
anti-PDPN Ab only
anti-PDPN NIR-PIT

Figure S3: Anti-PDPN NIR-PIT depletes PDPN+ cells in vitro but did not suppress tumor growth in vivo.

(A). Representative flow cytometry data of PDPN expression in NIH3T3 cells stimulated with TGF-β, gated on live cells (left) and a summary of PDPN induction induced by 20 ng/ml TGF-β in vitro. (n=3). *: $p<0.05$ by two-tailed Student's t-test.

(B) Flow cytometry analysis of TGF-β-stimulated NIH3T3 cells without (left) and with (middle) in vitro anti-PDPN NIR-PIT at 1 h., gated on PDPN $^{+}$ cells. Representative data (left, 50J NIR-PIT) and cumulative data (right) are shown (n=3). **: $p<0.01$ by one way ANOVA.

(C). Flow cytometry analysis of PDPN expression (gated on live cells) within MC38, LL/2, and MOC2 tumors. MOC2 cells in vitro did not express PDPN. Representative data of 3 replicates.

(D). Representative (n= 3) immunofluorescence image (bottom) and generated heat map (top) of PDPN expression (green) in the MOC2 tumor. Nuclei were counterstained with DAPI (blue).

(E). Tumor growth curve for MOC2 tumor experiment. Experimental group mice were untreated (control), or received an intravenous injection of either unconjugated anti-PDPN antibody (Ab only) or anti-PDPN IR700 conjugate followed by NIR-PIT at 50J 24h later (anti-PDPN NIR-PIT). Data presented as mean ± SEM. ns: not significant by one-way ANOVA. n=4-6 per group.

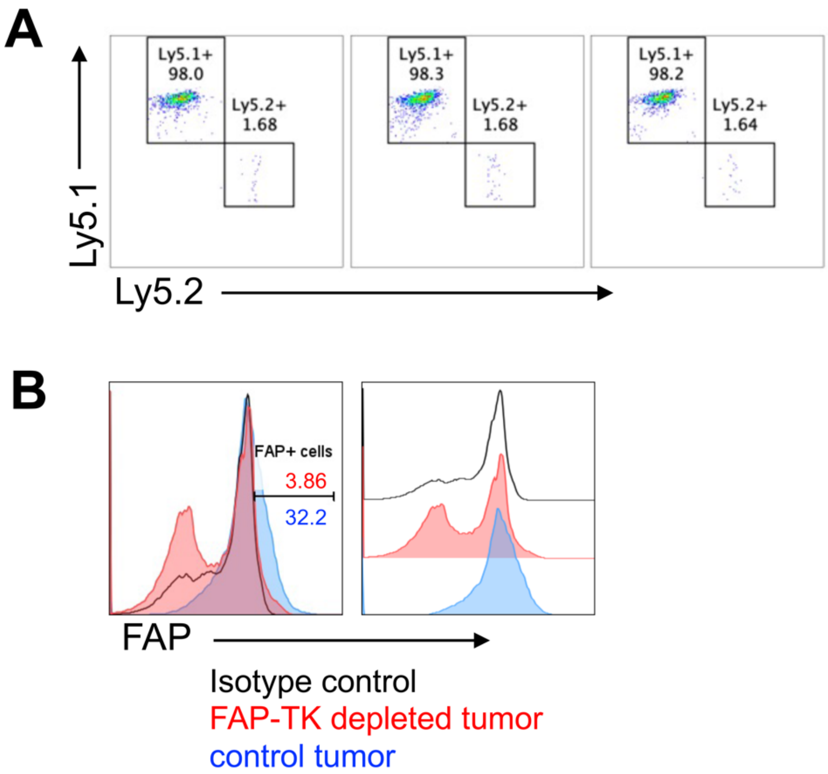

Figure S4: Validation of bone marrow chimera and depletion of FAP+ cells in tumor in FAP-TK mice.

(A). Chimera recipients were confirmed to have their peripheral blood cells >95% reconstituted with donor marrow-derived cells (Ly5.1) in FAP-TK recipients (Ly5.2) using flow cytometry analysis. Peripheral blood samples were analyzed at least six weeks after bone marrow transfer. Data represent 6-10 replicates.

(B). Representative flow cytometry data showed depletion of FAP^{+} cells in LL/2 tumor by GCV administration in FAP-TK mice (gated on live cells; red) compared with untreated control tumor (blue). Isotype control staining is shown in black line. Overlay (left) and half-offset (right) presentation of histograms. Data represents 3 replicates.
